# Effects of Six Weeks of High-Intensity Functional Training on Physical Performance in Participants with Different Training Volumes and Frequencies

**DOI:** 10.3390/ijerph17176058

**Published:** 2020-08-20

**Authors:** Rômulo Vasconcelos Teixeira, Gilmário Ricarte Batista, Arnaldo Luis Mortatti, Paulo Moreira Silva Dantas, Breno Guilherme de Araújo Tinôco Cabral

**Affiliations:** 1 Graduate Program on Physical Education, Federal University of Rio Grande do Norte, Natal, Rio Grande do Norte 59072970, Brazil; amortatti@gmail.com (A.L.M.); pgdantas@icloud.com (P.M.S.D.); brenotcabral@gmail.com (B.G.d.A.T.C.); 2 Department of Physical Education, Federal University of Rio Grande do Norte, Natal, Rio Grande do Norte 59072970, Brazil; 3 Department of Physical Education, Federal University of Paraíba, João Pessoa, Paraíba 58051900, Brazil; cajagr@gmail.com

**Keywords:** perceived exertion, injury, physical training, workload, exercise

## Abstract

High-intensity functional training (HIFT) is characterized by presenting high volumes and training intensities with constantly varied exercises. The aim of this study was to analyze the internal training load and the effects of high-intensity functional training on physical performance in subjects with different training volumes and frequencies. A total of 31 volunteers involved in high-intensity functional training (14 men and 17 women) were divided according to their training volumes and frequencies (high training-volume and frequency—HTVF; (*n* = 17) (nine women and eight men; age: 31.0 ± 6.3 years; height: 168.8 ± 8.1 cm, body weight: 73.6 ± 11.9 kg; BMI: 25.96 kg/m^2^) and moderate training volume and frequency—MTVF; (*n* = 14) (eight women and six men; age: 26.6 ± 4.7 years; height: 167.2 ± 8.6 cm, body weight: 75.8 ± 18.0 kg; BMI: 27.33 kg/m^2^)). The internal training load was determined using the session-rating of perceived exertion method. The monotony index (MI) and training strain (TS) were used to determine training variability during the training weeks. Countermovement vertical jump height, 20-m sprinting and handgrip strength were assessed at baseline and after six weeks of training. There was a time effect for MI ((F_(5, 145)_ = 5.942; *p* = 0.0001)), TS ((F_(5, 145)_ = 5.734; *p* = 0.0001)), weekly internal training load ((F_(4.006, 116.87)_ = 4.188; *p* = 0.003)) and mean weekly internal training load ((F_(4.006, 116.87)_ = 4.188; *p* = 0.003)). There was no increase in performance in either group for countermovement vertical jump height ((F_(1,29)_ = 6.081; *p* = 0.050)), sprinting ((F_(1,29)_ = 1.014; *p* = 0.322)), right handgrip strength ((F_(1,29)_ = 2.522; *p* = 0.123)) or left handgrip strength ((F_(1,29)_ = 2.550; *p* = 0.121)). The current findings suggest that six weeks of high-intensity functional training was not able to increase performance in either group. Therefore, different volumes and frequencies do not seem to influence the increase in physical performance of HIFT practitioners.

## 1. Introduction

High-intensity functional training (HIFT) is a modality characterized by presenting high volumes and training intensities [1] with constantly varied exercises [2] with or without any recovery interval between the series [3]. HIFT training sessions consist of Olympic weightlifting exercises (OWE) (e.g., clean and jerk, snatch), gymnastics (e.g., lunges and pull-ups) and metabolic conditioning (e.g., running and rowing) [2]. In addition to the diversity of functional movements performed in high intensity, HIFT aims to improve physical conditioning variables (i.e., strength, body composition, among others) and performance (i.e., speed, power, among others) [4]. Thus, HIFT has gained status in sports in recent years and investigations have consequently emerged around physical capabilities for success in the HIFT [5,6].

There are several determinants for success in sports such as vertical jumping [7], sprinting [8] and handgrip strength (HS) [9]. For example, the presence of short-haul running races (i.e., up to 800 m) which are constantly held in HIFT competitions are associated with success in HIFT [5]. In addition, the scientific literature has suggested that the training volume appears to be a determining factor in the magnitude of strength gains [10,11]. For example, it was observed that training protocols with equalized volume and different training frequencies (i.e., six times vs. three times) do not differ for gains in maximum strength in the bench press after six weeks of training [12]. Additionally, Saric et al. [13] did not observe differences between groups for strength gains in the upper and lower limbs in using a similar methodology and training time. Finally, Gomes et al. [14] analyzed the influence of an equalized and progressive training volume in highly trained men (i.e., ~7 years of experience). It was observed that there were no differences between groups for strength gains in the bench press and squat exercises after eight weeks of training. Although HIFT incorporates these capacities (i.e., vertical jumping, sprinting and handgrip strength) and high volumes and intensities of training, no studies to date have examined the impact of different volumes and training frequencies of HIFT on physical performance. This issue can be of great relevance for coaches and can give light to the science of HIFT.

Thus, understanding the influence of HIFT volume and training frequency can provide an efficient prescription with overload which enables adequate adaptation with less interference in the health status of practitioners, as it has been demonstrated that two consecutive days of HIFT may promote possible immunosuppression [15] and that training protocols with high volumes and absence of recovery intervals promote increased damage markers (i.e., creatinokinase and interleukin-6) [16]. In addition, training load management is of fundamental importance for performance enhancement [17], since long-term accumulated training loads, intensification periods and acute changes in the training load have been identified as potential causes for loss in performance [18], diseases and injuries [19]. On the other hand, it was observed that six months of HIFT is able to promote positive chronic responses in the immune and hormonal system [20]. Therefore, monitoring the internal training load (ITL) becomes necessary to better adapt to training along with minimizing the risk of injury [17].

The internal training load (ITL) reports the effects of loads on the body experienced by an athlete after training [21]. Due to the diversity of exercises applied in HIFT (i.e., gymnastics, strength and metabolic conditioning), the management of training loads is a challenge for coaches [17] who need efficient tools and with practical applicability. There are countless tools for controlling stress/recovery from training, such as creatine kinase, testosterone/cortisol ratio and immunological markers [22]. However, these tools are expensive, invasive and are not commonly used in daily training practice [17]. Thus, coaches need tools that reproduce the “real world” [17] and are effective and sensitive to variations in workloads, such as the session rating of perceived exertion (session-RPE) [23].

The session-RPE is one of the most used tools in sports for monitoring ITL [24,25] because it provides information about the physiological stress imposed by the training process [18]. The ITL control method based on session-RPE generates other important variables such as monotony index (MI) and training strain (TS) which can indicate training overload [26]. These indices were recently validated for HIFT [27,28], showing to be efficient with regard to ITL differentiation in different training phases [29]. Therefore, it is of fundamental importance to use efficient and validated tools in order to individualize the training and take into account the level of each subject.

Despite the exponential growth of both the modality and number of practitioners, there is a limited number of studies related to safety in HIFT practitioners. Studies have not performed ITL quantification or monitoring in order to analyze the stress/recovery ratio and the performance determinants for the sport [3] as measured through the session-RPE, MI and TS, nor a verification of physical performance between subjects with different training volumes and frequencies through performance tests after a 6-week training period. Hence, to our knowledge, this is the first study to examine the impact of different HIFT volumes and training frequencies on physical performance.

Thus, the aim of this study was to analyze the ITL during six weeks of HIFT and to verify the effects of HIFT on physical performance in subjects with different training volumes and frequencies. The hypothesis of the study is that the six weeks of HIFT may generate different ITL with increased physical performance, regardless of the training volumes and frequencies presented.

## 2. Materials and Methods

### Participants

The study design is characterized as observational. An a priori power analysis was computed using G*Power 3.1.9.4. Thus, a significant variable from a previous study (i.e., back squatting) was used for presenting improvement after performing HIFT [30]. The lowest significant effect size of back squatting (η^2^p = 0.55) was used for the power analysis. Thus, a sample size of 10 subjects in each group was calculated by inputting 0.55 as the effect size and setting the alpha significance level at 0.05 and power to 0.80. A total of 31 subjects (14 men and 17 women) of different training volumes and frequencies (high training-volume and frequency—HTVF (27.9 ± 9.2 months of training background), *n* = 17 (9 women and 8 men; age: 31.0 ± 6.3 years; height: 168.8 ± 8.1 cm, body weight: 73.6 ± 11.9 kg; BMI: 25.96 kg/m^2^) and moderate training volume and frequency—MTVF (8.3 ± 3.7 months of training background), *n* = 14 (8 women and 6 men; age: 26.6 ± 4.7 years; height: 167.2 ± 8.6 cm, body weight: 75.8 ± 18.0 kg; BMI: 27.33 kg/m^2^)) were followed for 6 weeks. The subjects were grouped by their training volumes and frequencies into HTVF and MTVF groups. The weekly frequency and training session time was configured into the following groups: HTVF (5 to 6 days; ~2 h) and MTVF (3 to 4 days; ~1 h). It is worth noting that all subjects participated in all the procedures inherent to the study, and therefore there was no sample loss. The project was explained to all subjects of the training center and those who volunteered to participate signed the free and informed consent form. The researchers spent a week at the training center’s facilities explaining the study and aiming to recruit as many subjects as possible. The following inclusion criteria were adopted: (i) presenting a weekly minimum training frequency of 3 times; and (ii) over 18 years of age. All those with osteomioarticular injuries and who did not meet at least 75% of the training sessions were excluded from the sample. The adherence rate during the 6 weeks was 82.4% for the HTVF group and 84.3% for the MTVF group.

The study was approved by the local ethics research committee (No. 3,082,357) and followed all of the ethical standards set forth in the Helsinki Declaration 2013 (and the World Medical Association).

## 3. Procedures

### Training Sessions

The subjects were familiar with all the adopted procedures and tests which were usually used during their training program. The schedule and training organization during the collection period were structured and programmed by the coaches responsible for the training center in order to provide a control between stress and recovery, thereby enabling subjects to handle the physical and physiological demands well throughout the collection period. All experimental procedures of the performance tests were performed at the beginning of each week (i.e., baseline and post 6 weeks). The subjects were instructed to maintain their usual diet and refrain from alcohol, caffeine and high-intensity exercise in the 72h preceding the test performance. In addition, all tests (i.e., countermovement vertical jump height (CVJH), sprints and handgrip strength (HS)) were performed indoors and at the same time of day to mitigate the climatic effects. The subjects were also submitted to a warm-up protocol proposed by the training center coaches for approximately 10 min prior to the tests consisting of different running speeds, jumps and specific HIFT activity. The subjects initially performed the CVJH, followed by sprints and HS.

The training sessions usually began with a general warm-up (i.e., squats, multi-joint exercises, among others) (~20 min for HTVF and ~10 min for MTVF). After the warm-up, the training session consisted of movement technique exercises and strength training (i.e., Olympic weightlifting, squats, bench-press, deadlift and their variations) (~40 min for HTVF and ~20 min for MTVF), and finally gymnastic exercises (hand stands, bar exercises, ring, among others) and metabolic conditioning (rowing, races, among others) (~60 min for HTVF and ~30 min for MTVF). The objective of the conditioning sessions was to conclude them in the shortest time possible, while the other conditioning sessions on later days (depending on the planning/organization of the weekly training) were intended to perform the highest number of repetitions within the subject’s limit in a fixed time period. All subjects performed the same training program, however, the HTVF group performed higher training volumes and frequencies (i.e., higher weekly frequency and training session time), as mentioned above. In addition, as the individual capacity (for example, relative strength or ability to perform specific exercises) among subjects were distinct, all the loads and exercises were individualized (i.e., the load and exercises were modified by the coach when necessary so that each subject, regardless of the training volumes and frequencies, could complete all the proposed tasks). It is worth mentioning that the determination of the external training load was not carried out due to the modality characteristic which presents a wide variety of exercises in the same training session (i.e., strength, gymnastics and endurance), therefore constituting a challenge for coaches and future research [17]. However, monitoring was performed minimally through the session time.

The monitoring lasted 6 weeks with performance tests (i.e., CVJH, sprints and HS) before and after the training period. In addition, the session-RPE was calculated (i.e., RPE x session time) after each training session over the 6 weeks [26].

## 4. Instruments

### Quantifying Training Load

The ITL was recorded using the session-RPE rating of perceived exertion scale [26]. The intensities of the training sessions were similar between the groups and proposed by the coach of the training center. However, according to the principle of individuality, each subject could respond differently to the intensity proposed by the coach. The subjects were asked how intense their training session was at around ~30 min after the end of each training session and responded based on a category ratio scale (CR-10) [26]. The subjects were already previously familiar with the CR-10 scale. The reported value was multiplied by the total duration of each training session in minutes, resulting in an arbitrary unit value (AU) (perceived internal training load) [26]. The training load was expressed in weekly internal training load (WITL) (the sum of 7 days). The mean weekly ITL (WMITL) was additionally performed by the sum of the weekly AU divided by 7 (number of days of the week). Finally, the session-RPE values (daily and weekly) were used for the analyses. The MI was calculated by the division of the WMITL by its respective standard deviation [26]. The TS was calculated by multiplying the sum of the weekly ITL (WITL) by MI of the same time interval [26].

## 5. Physical Performance

### 5.1. Countermovement Vertical Jump Height (CVJH)

The vertical jump height was verified from the use of CVJH. The subjects were familiar with performing the jump test and able to exert a downward movement followed by a complete extension of the legs and were free to establish the amplitude of the countermovement to avoid modifications in the jumps [31]. All attempts were performed with their hands fixed on their hips and subjects were encouraged to jump as high and as fast as possible [31]. A contact platform connected to the Jump Test Pro 2.10 software program (Cefise^®^, São Paulo, Brazil) was used to measure CVJH, and five attempts with intervals of 15 s between them were granted [32]. Finally, the jump was considered valid for analysis if the takeoff and landing positions remained visually analogous. The best result of five attempts was subsequently used for data analysis. The intraclass correlation coefficient (ICCs) of the baseline and post CVJH were 0.99 in both.

### 5.2. Speed Test (20-m Sprint)

Prior to the speed tests and after the warm-up period proposed by the coach responsible for the training center, 1 photocell (Cefise^®^, São Paulo, Brazil) was allocated at 20 m from the starting point. A 20-m test was repeated (i.e., two times) from the standing position. All speed tests were performed at an indoor training center in order to mitigate weather effects. Recovery time between trials was 5 min and the best time was used for data analysis [32]. The ICCs of the baseline and post times of 0–20 m were 0.96 and 0.95, respectively.

### 5.3. Handgrip Strength (HS)

The subject was comfortably seated, positioned with their shoulder slightly adducted, elbow flexed at 90°, forearm in neutral position and the wrist positioning could oscillate from 0° to 30° long. These procedures are in accordance with the specifications of the American Society of Hand Therapists [33]. The dynamometer measurement (Jamar^®^, São Paulo, Brazil) was regulated according to the characteristics of each subject and the best results of three attempts in each hand were used for data analysis. ICCs were 0.99 for right HS (baseline and post) and 0.98 for left HS (baseline and post).

### 5.4. Statistical Analyses

Normality was tested using the Shapiro–Wilk test and Z-score analysis of asymmetry and kurtosis (−1.96 to 1.96). Continuous data are reported in mean and standard deviation. A mixed ANOVA of repeated measures (2 conditions and 6 times) was used to verify the MI, TS and magnitude of WITL and WMITL behavior within and between the different training volumes and frequencies (HTVF and MTVF) over 6 weeks of HIFT. A mixed ANOVA of repeated measures (2 conditions and 2 times) was adopted to verify differences in performance within and between training volumes and frequencies (HTVF and MTVF). Mauchly’s sphericity test was adopted to verify the data measurement. The partial eta squared (η^2^p) effect size (ES) was used for mixed ANOVA of repeated measures, being classified as: no effect (ES: < 0.04), minimum effect (ES: 0.04–0.25), moderate effect (ES: 0.25–0.64) and strong effect (ES: > 0.64) [34]. Bonferroni’s post hoc was used to identify specific differences. A significance level of *p* < 0.05 was adopted for all analyses.

## 6. Results

An interaction effect was verified for MI ((F_(5, 145)_ = 2.912; *p* = 0.016; η^2^p = 0.091; power = 0.839, minimum effect)) and TS ((F_(5, 145)_ = 2.810; *p* = 0.019; η^2^p = 0.088; power = 0.824, minimum effect)). However, no interaction effect was verified for WITL ((F_(4.006, 116.187)_ = 1.855; *p* = 0.123; η^2^p = 0.060; power = 0.549, minimum effect)) or WMITL ((F_(4.006, 116.187)_ = 1.855; *p* = 0.123; η^2^p = 0.060; power = 0.549, minimum effect)). There was a time effect for MI ((F_(5, 145)_ = 5.942; *p* = 0.0001; η^2^p = 0.170; power = 0.994, minimum effect)), TS ((F_(5, 145)_ = 5.734; *p* = 0.0001; η^2^p = 0.165; power = 0.992, minimum effect)), WITL ((F_(4.006, 116.87)_ = 4.188; *p* = 0.003; η^2^p = 0.126; power = 0.914, minimum effect)) and WMITL ((F_(4.006, 116.87)_ = 4.188; *p* = 0.003; η^2^p = 0.126; power = 0.914, minimum effect)). There was only a difference in the MTVF group for MI from week 1 to week 4 (∆% = 0.416; CI 95% = 0.097 to 0.735; *p* = 0.004), from week 1 to week 5 (∆% = 0.551; CI 95% = 0.158 to 0.944; *p* = 0.002), from week 3 to week 4 (∆% = 0.232; CI 95% = 0.020 to 0.444; *p* = 0.022) and between weeks 3 and 5 (∆% = 0.367; CI 95% = 0.054 to 0.680; *p* = 0.012). There was only a difference in the MTVF group for TS from week 1 to week 4 (∆% = 1476.3; CI 95% = 316.6 to 2635.9; *p* = 0.005), from week 1 to week 5 (∆% = 1788.9; CI 95% = 477.8 to 3099.9; *p* = 0.002), from week 3 to week 4 (∆% = 1023.1; CI 95% = 170.2 to 1875.9; *p* = 0.009) and between weeks 3 and 5 (∆% = 1335.7; CI 95% = 225.0 to 2446.3; *p* = 0.009). There was only a difference in the MTVF group for WITL from week 3 to week 4 (∆% = 558.9; CI 95% = 198.4 to 919.3; *p* = 0.0001) and between weeks 3 and 5 (∆% = 630.7; CI 95% = 6.1 to 1255.2; *p* = 0.046). There was only a difference in the MTVF group for WMITL from week 3 to week 4 (∆% = 79.8; CI 95% = 28.3 to 131.3; *p* = 0.0001) and between weeks 3 and 5 (∆% = 90.0; CI 95% = 0.8 to 179.3; *p* = 0.046). No group effect was verified for MI ((F_(1,29)_ = 0.095; *p* = 0.760; η^2^p = 0.003; power = 0.060, no effect)), TS ((F_(1,29)_ = 0.029; *p* = 0.865; η^2^p = 0.001; power = 0.053, no effect)), WITL ((F_(1,29)_ = 0.094; *p* = 0.761; η^2^p = 0.003; power = 0.060, no effect)) and WMITL ((F_(1,29)_ = 0.094; *p* = 0.761; η^2^p = 0.003; power = 0.060, no effect)) (see Table 1).

**Figure 1:**
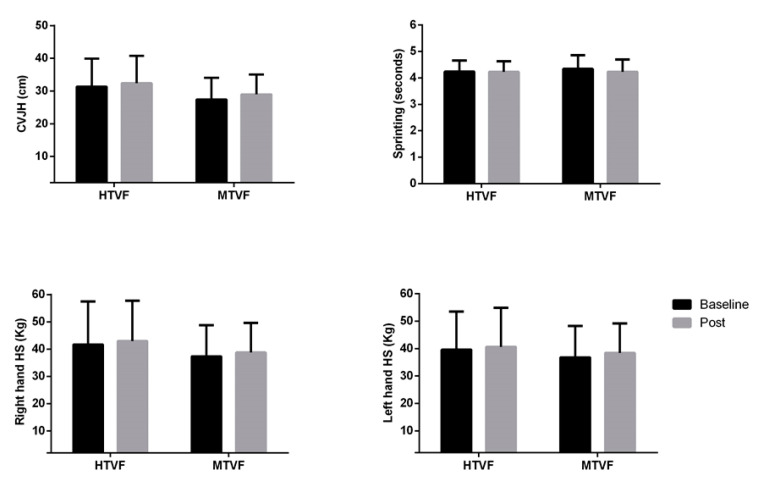
Countermovement vertical jump height (CVJH), sprinting, right and left handgrip strength between groups in HIFT. HTVF—high training-volume and frequency; MTVF—moderate training volume and frequency.

No interaction effect was verified for CVJH ((F_(1,29)_ = 0.232; *p* = 0.634; η^2^p = 0.008; power = 0.075, no effect)), sprinting ((F_(1,29)_ = 0.679; *p* = 0.417; η^2^p = 0.023; power = 0.125, no effect)), right HS ((F_(1,29)_ = 0.013; *p* = 0.909; η^2^p = 0.0001; power = 0.051, no effect)) or left HS ((F_(1,29)_ = 0.126; *p* = 0.725; η^2^p = 0.004; power = 0.064, no effect)). No time effect was verified for CVJH ((F_(1,29)_ = 6.081; *p* = 0.050; η^2^p = 0.173; power = 0.664, minimum effect)), sprinting ((F_(1,29)_ = 1.014; *p* = 0.322; η^2^p = 0.034; power = 0.164, no effect)), right HS ((F_(1,29)_ = 2.522; *p* = 0.123; η^2^p = 0.080; power = 0.336, minimum effect)) or left HS ((F_(1,29)_ = 2.550; *p* = 0.121; η^2^p = 0.081; power = 0.339, minimum effect)). No group effect was verified for CVJH ((F_(1,29)_ = 1.865; *p* = 0.183; η^2^p = 0.060; power = 0.262, minimum effect)), sprinting ((F_(1,29)_ = 0.085; *p* = 0.773; η^2^p = 0.003; power = 0.059, no effect)), right HS ((F_(1,29)_ = 0.771; *p* = 0.387; η^2^p = 0.026; power = 0.136, no effect)) or left HS ((F_(1,29)_ = 0.301; *p* = 0.587; η^2^p = 0.010; power = 0.083, no effect)) (see Figure 1).

**Figure 2:**
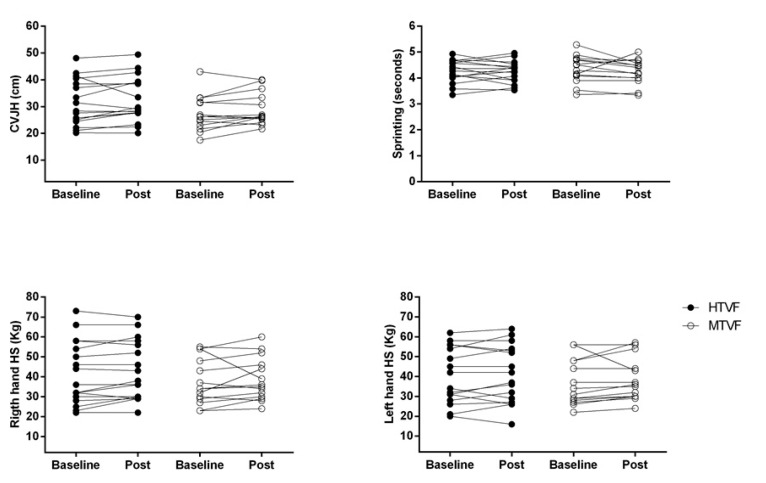
Individual values for countermovement vertical jump height (CVJH), sprinting, right and left handgrip strength between groups in HIFT. HTVF—high training-volume and frequency; MTVF—moderate training volume and frequency.

Figure 2 reports the individual values for the performance tests after 6 weeks of HIFT between groups. It was observed that 70.58% (i.e., 12 subjects) and 64.28% (i.e., 9 subjects) increased performance for the CVJH, 52.94% (i.e., 9 subjects) and 71.42% (i.e., 10 subjects) for sprinting, 41.17% (i.e., 7 subjects) and 71.42% (i.e., 10 subjects) for right HS and 52.94% (i.e., 9 subjects) and 71.42% (i.e., 10 subjects) for left HS of the HTVF group and of the MTVF group, respectively, after 6 weeks, although not significant.

## 7. Discussion

The aim of this study was to analyze the ITL during six weeks of HIFT and to verify the effects of HIFT on physical performance in subjects with different training volumes and frequencies. No differences in MI, TS, WITL and WMITL in the HTVF group were observed over the six weeks. On the other hand, a variation of greater magnitude in MI, TS, WITL and WMITL in the MTVF group was observed over the six weeks (see Table 1). Regarding physical performance, six weeks of high-intensity functional training was not able to generally increase performance in either group (see Figure 1).

The arrangement of WITL and WMITL only differed during the six weeks of training in the MTVF group. The American College of Sports Medicine recently published a consensus on HIFT and suggested monitoring the training load to mitigate negative adaptations [35], since high training loads associated with low recovery is one of the main causes of overtraining [36]. These studies showed the relevance of applying a floating approach and periodic control of workloads for positive development of performance [37]. Thus, the willingness and adjustments of workloads in accordance with the state of readiness of subjects is shown to be a valuable strategy for efficient and safe prescription [38,39] in order to prevent detraining and promote increased performance [40].

Our data show a WMITL of 1903 AU for the HTVF group and 1967 AU for the MTVF group. The ITL values are in line with the values found by Tibana et al. [17] (2092 AU) and slightly below the values presented by Williams et al. [29] (2591 AU) who used session-RPE to quantify training loads in HIFT athletes. It is noteworthy that periods of load intensification, long-term accumulated loads, as well as strenuous competitions are possible predictors of injuries [19]. For example, weekly workloads between 3000 and 5000 AU revealed 50% to 80% more chances of injuries [41], as well as two subsequent weeks with weekly workloads greater than 2000 AU [42]. On the other hand, weekly workloads below 1250 AU also revealed potentiation in the risk of injury and did not promote increased fitness [42]. Therefore, session-RPE can assist coaches in performing “adjustments” of training loads when necessary, respecting the individuality of subjects, and in order to avoid high MI and TS [26] which constitute factors associated with larger chances of injury.

It is worth mentioning that no injuries were observed during the monitoring period in our study. This condition was consistent with the MI and TS values which were below the values that indicate a higher risk of injury or illness in both groups (see Table 1). Abrupt growth stemming from MI (i.e., above 2.0 AU) as well as high TS (i.e., above 8000 AU) were correlated with 77% and 89% in the occurrence of diseases, respectively [26]. In addition, high volumes and training intensities may have repercussions on the decline of the immune system and consequently increases in signs and symptoms of upper respiratory tract infection [43]. For example, Ferrari et al. [44] observed significant associations between upper respiratory tract infection and TS in the preparatory (r = 0.72; *p* = 0.03) and competitive phases (r = 0.73; *p* = 0.03) in trained cyclists. On the other hand, it was observed that six months of HIFT is able to promote positive chronic responses in the immune and hormonal systems [20]. In addition, performance tests are necessary for correct management of training loads and should be used in order to avoid non-functional overreaching and consequently overtraining [36].

Considering variations in training loads, it was observed that six weeks of high-intensity functional training was not able to generally increase performance in either group in our study. The literature suggests a dose–response relationship for training volume and increased performance [10,11]. For example, Brigatto et al. [45] compared different training volumes (i.e., number of sets) in strength gains in trained subjects (i.e., ~4 years of experience). The results suggest greater strength gains for the group which performed the highest training volumes. On the other hand, Jeffreys et al. [46] evaluated the effects on the performance of high volume protocols (i.e., 1920 ground contacts) vs. low volume (i.e., 480 ground contacts) of plyometric training in rugby players. It was observed that both groups increased performance in a similar way. However, the group with low training volume performed 75% less training volume. In addition, previous experience and the previously used training volume seem to be determining factors in decision making during the prescription of the training volume [47]. For example, Scarpelli et al. [47] compared standardized volumes (i.e., based on literature studies) vs. individualized volumes (i.e., based on previous experience). It was observed that muscle hypertrophy showed higher magnitudes for the group with individualized training volume (~10% vs. ~6%). Therefore, it is of fundamental importance that the principle of individuality be taken into account for assertive decision making in the training prescription.

CVJH has been widely adopted in order to verify adaptations and neuromuscular fatigue in relation to training [48]. Thus, training loads can be defined according to the vertical jump height oscillations and can be applied in training sessions (i.e., daily) with the objective of evaluating and effectively controlling neuromuscular responses and the readiness state of the subjects [31]. Studies have recently indicated associations between olympic weightlifting movements which are commonly used in HIFT [2] and increases in vertical jump height [49] and muscle power [50]. Increased muscle power provides valuable evolution in performance in sports [32]. Previous studies have shown that performing HIFT presents potential for maximizing performance [51,52], possibly due to the use of complex training (i.e., strength training paired with power exercises) [53], which in turn provides increases in vertical jump and sprint responses; however, the training program duration and subjects’ profile should be considered [54].

The results regarding the sprinting test showed that there is no change in performance across the training period or among groups in general. Sprinting speed is of great importance for achieving success in sports [55]. For example, the presence of short-haul running races (i.e., up to 800 m) which are constantly held in HIFT competitions are associated with success in the sport [5]. Strong correlations were additionally observed between the best classifications of female athletes and the performance of sprints of 50 and 400 m (r = 0.77 and 0.69, respectively) presented in an official HIFT competition [8]. Therefore, sprinting should not be overlooked in the subject’s training program in conjunction with maximum strength, especially when the modality requires these skills in training sessions and competitions [49]. Sprinting measured in the present study was short-distance (i.e., 20 m), which is considered the initial phase of acceleration [56]. Thus, it is possible that distinct results could have been found at greater distances.

There were no general differences verified in relation to handgrip strength (HS) after the 6-weeks of HIFT between groups. HS is of great importance in sports which require higher levels of HS to maximize performance and injury prevention [57]. Olympic weightlifting and gymnastics modalities [57] stand out among different sports which are often employed in HIFT [2]. For example, a varied relationship (r = 0.81; *p* < 0.05) between HS and endurance HS was observed in ring athletes [58]. Thus, high levels of HS are valuable for successful achievement in modalities which present exercises in rings and bars [57]. In addition, coaches need assessment tools that are sensitive, easy to handle and which replicate the sports movements, such as HS [57]. Therefore, HS can provide relevant information in order to identify possible changes in performance or during different periods of the rehabilitation process [9].

Although the results of this study are of great importance for coaches and sports scientists, some limitations need to be highlighted: (i) The external training load was not determined due to the modality characteristic which presents a wide variety of exercises in the same training session (i.e., strength, gymnastics and endurance), therefore constituting a challenge for coaches and future research [17]. However, monitoring was performed minimally through the session time; (ii) The absence of tapering (i.e., volume reduction with maintenance or increased training intensity); however, it is worth mentioning that the training was proposed by the training center coach, making it impossible to intervene in the prescription and training organization. On the other hand, all subjects were instructed to refrain from high-intensity exercise in the 72 h preceding the test performance. Nevertheless, this is the first study to examine the impact of different training volumes and frequencies of HIFT on physical performance. Therefore, further studies should be conducted to examine the impact of different training volumes and frequencies of HIFT with the inclusion of tapering and monitoring the external training load.

From a practical point of view, the present results indicate that coaches, sports scientists and other professionals in the field should manage workloads during the season with the aim to adjust and opt for consistent workloads respecting the individuality of subjects. Thus, tools which are accessible, efficient and non-invasive such as session-RPE provide simple and objective results on workloads and additionally on the readiness state of subjects. Therefore, daily and weekly controls can collaborate with the training planning and organization in order for athletes/subjects to reach the apex of physical form with lower risks of injury. Regarding performance tests, coaches can monitor adaptations in relation to the proposed training period and consequently opt for better training strategies in order to achieve superior results in training and subsequent competitions, since in our study it was observed that six weeks of high-intensity functional training was not able to increase performance, regardless of the training volume or frequency.

## 8. Conclusions

It was observed that the internal training load presented different magnitudes during the six weeks in both groups with differences only for the moderate training volume and frequency group. In addition, the current findings suggest that six weeks of high-intensity functional training were not able to increase performance in subjects with high training-volume and frequency or a moderate training volume and frequency. Therefore, coaches must opt for better training strategies in order to achieve superior results in training and subsequent competitions.

## Figures and Tables

**Table 1:** Monotony index, training strain, weekly internal training load (ITL) and mean weekly ITL.

Variables	Groups	Week 1	Week 2	Week 3	Week 4	Week 5	Week 6
MI	HTVF	1.12 ± 0.25	0.99 ± 0.46	1.03 ± 0.38	1.08 ± 0.30	0.94 ± 0.27	1.16 ± 0.46
	MTVF	1.33 ± 0.42 ^†,∞^	1.02 ± 0.30	1.15 ± 0.31 *^,$^	0.91 ± 0.22	0.78 ± 0.23	0.98 ± 0.18
TS	HTVF	2477.2 ± 1186.6	2072.3 ± 1653.1	2085.3 ± 1404.1	2310.5 ± 1310.3	1737.1 ± 911.3	2602.8 ± 1848.3
	MTVF	3143.4 ± 1422.2 ^†,∞^	2123.5 ± 1366.9	2690.2 ± 1175.9 *^,$^	1667.1 ± 887.4	1354.5 ± 797.2	1966.9 ± 622.7
WITL	HTVF	2118.2 ± 775.6	1764.7 ± 992.6	1830.2 ± 741.3	1980.4 ± 778.1	1729.1 ± 642.5	1994.4 ± 802.9
	MTVF	2283.9 ± 558.2	1889.2 ± 750.8	2281.4 ± 789.4 *^,$^	1722.5 ± 675.0	1650.7 ± 620.6	1973.9 ± 442.7
WMITL	HTVF	302.5 ± 110.8	252.1 ± 141.8	261.4 ± 105.9	282.9 ± 111.1	247.0 ± 91.7	284.9 ± 114.7
	MTVF	326.2 ± 79.7	269.8 ± 107.2	325.9 ± 112.7 *^,$^	246.0 ± 96.4	235.8 ± 88.6	281.9 ± 63.2

MI—monotony index; TS—training strain; WITL—weekly internal training load; WMITL—mean weekly internal training load; HTVF—high training-volume and frequency; MTVF—moderate training volume and frequency; ^†^—different from week 4 (*p* < 0.05); ^∞^—different from week 5 (*p* < 0.05); *—different from week 4 (*p* < 0.05); ^$^—different from week 5 (*p* < 0.05); ITL—internal training load.
